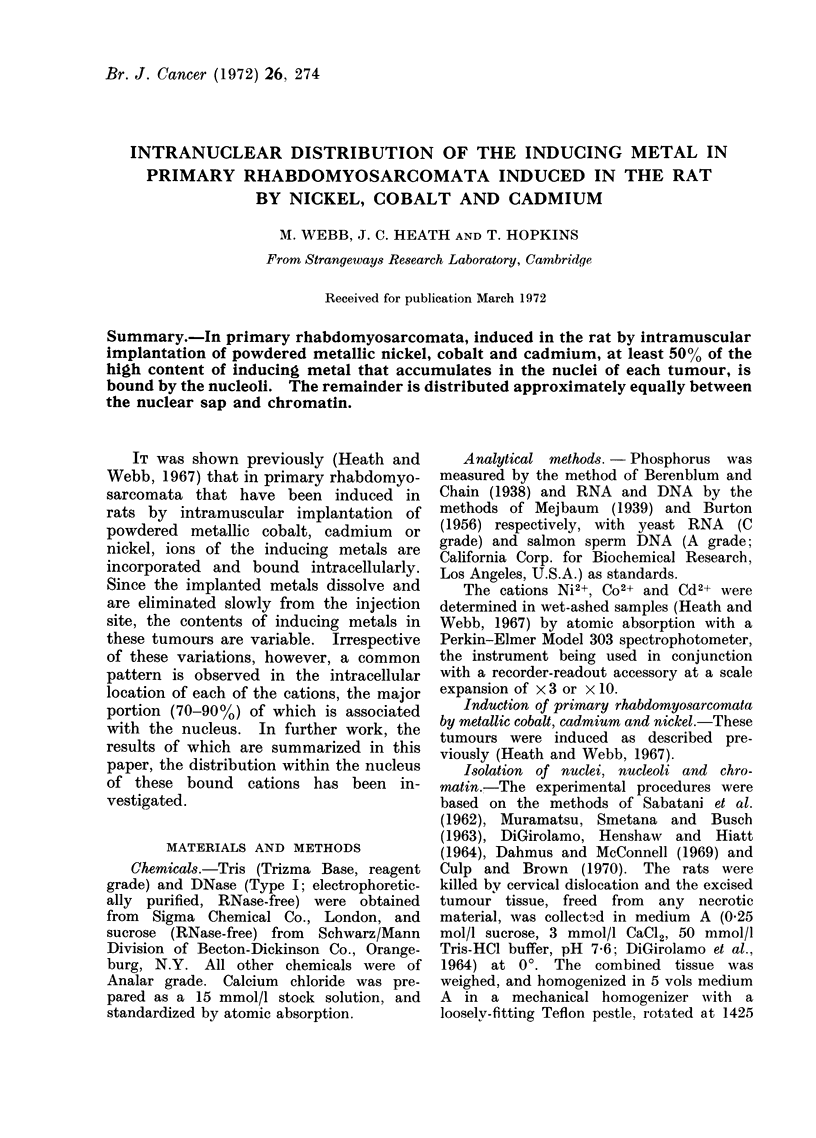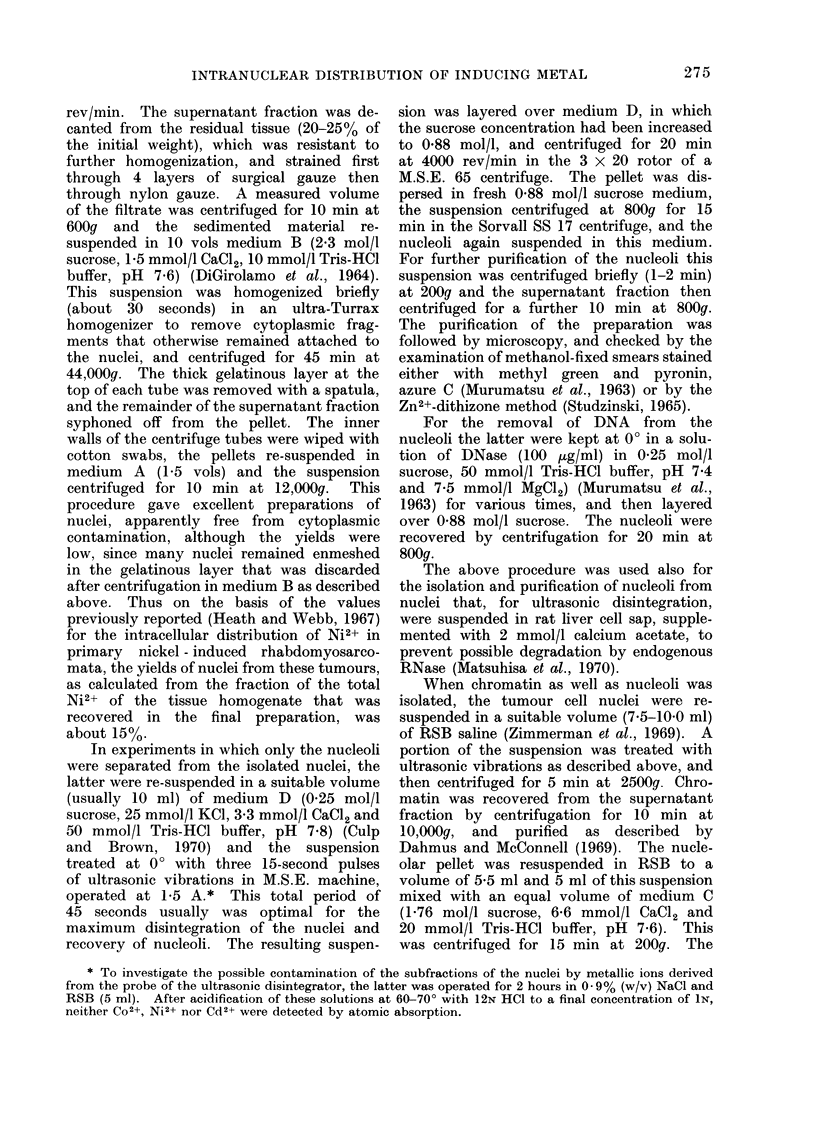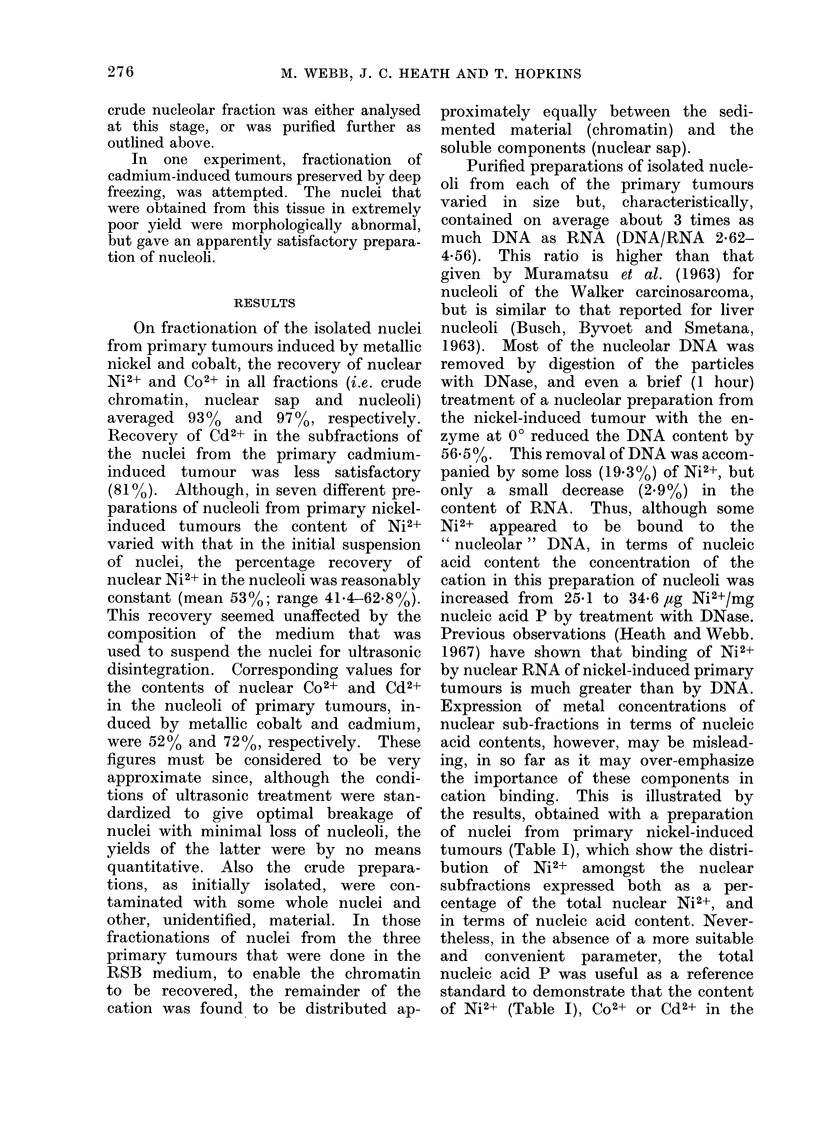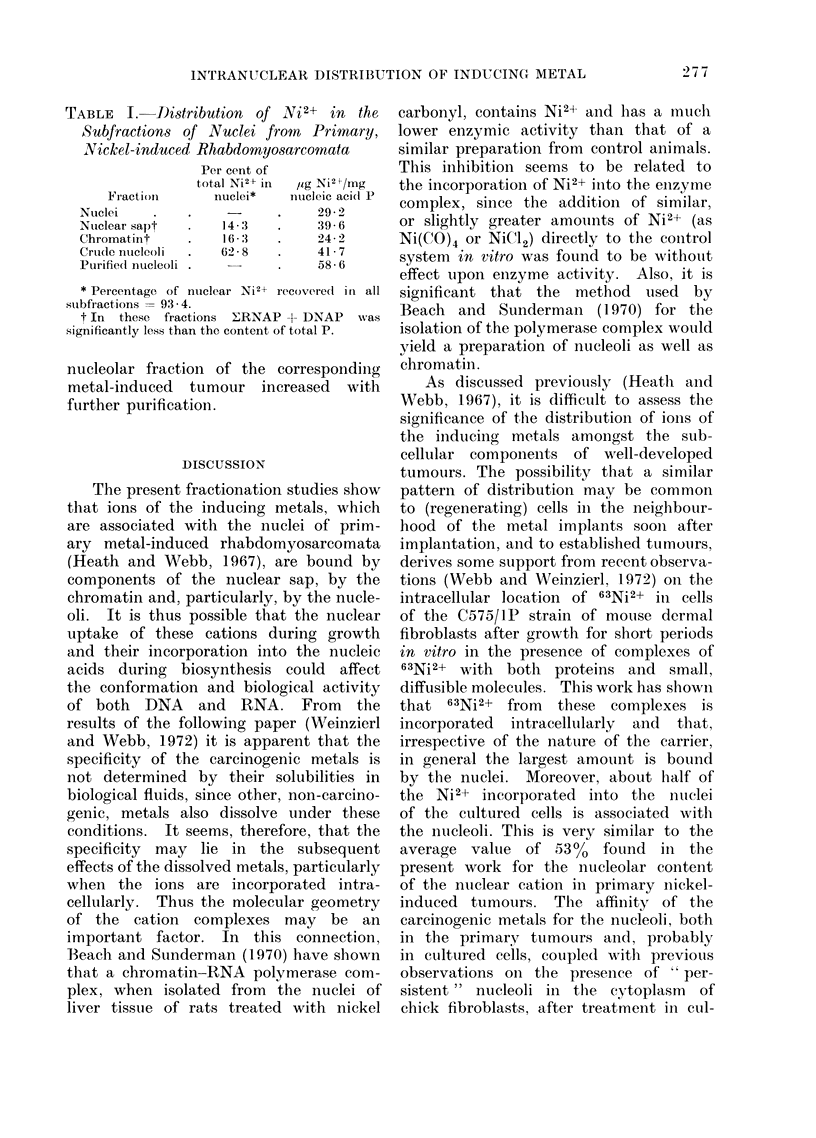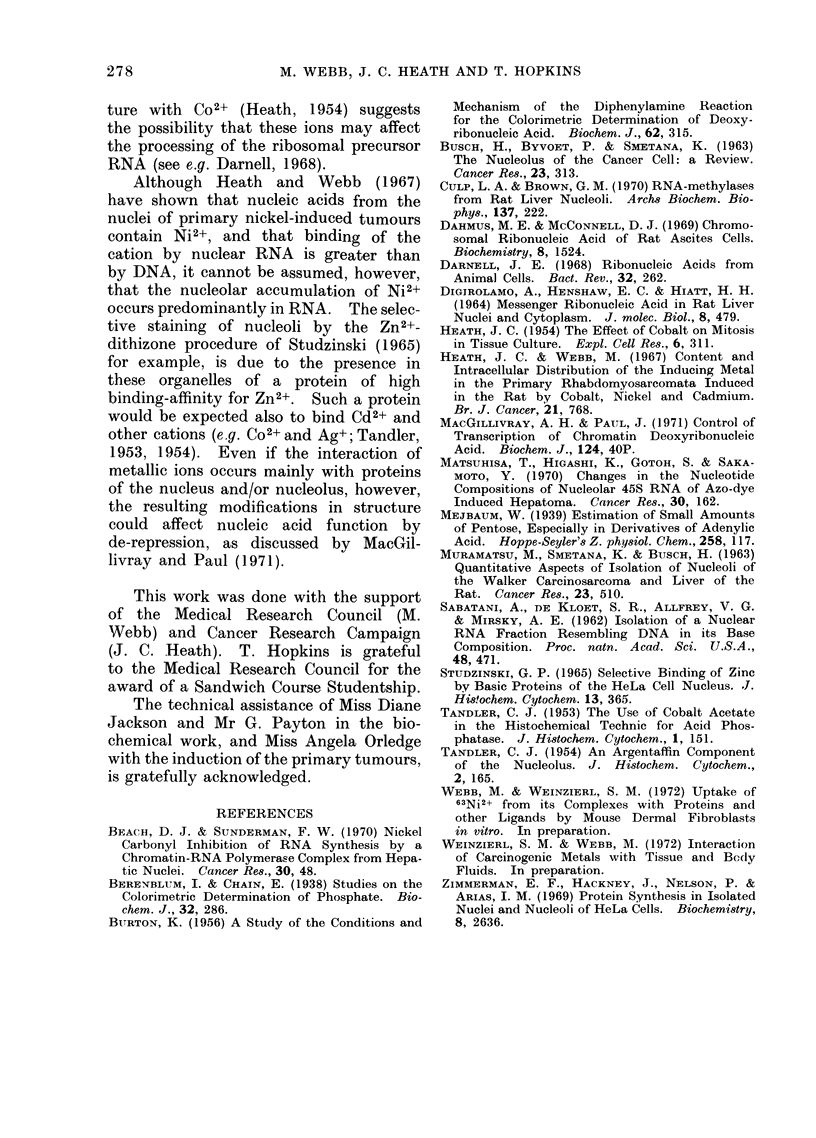# Intranuclear Distribution of the Inducing Metal in Primary Rhabdomyosarcomata induced in the Rat by Nickel, Cobalt and Cadmium

**DOI:** 10.1038/bjc.1972.37

**Published:** 1972-08

**Authors:** M. Webb, J. C. Heath, T. Hopkins

## Abstract

In primary rhabdomyosarcomata, induced in the rat by intramuscular implantation of powdered metallic nickel, cobalt and cadmium, at least 50% of the high content of inducing metal that accumulates in the nuclei of each tumour, is bound by the nucleoli. The remainder is distributed approximately equally between the nuclear sap and chromatin.


*Br. J. Cancer* (1972) **26**, 274

### INTRANUCLEAR DISTRIBUTION OF THE INDUCING METAL IN PRIMARY RHABDOMYOSARCOMATA INDUCED IN THE RAT BY NICKEL, COBALT AND CADMIUM

M. WEBB, J. C. HEATH AND T. HOPKINS

*From Strangeways Research Laboratory, Cambridge*

Received for publication March 1972

**Summary.—In primary rhabdomyosarcomata, induced in the rat by intramuscular implantation of powdered metallic nickel, cobalt and cadmium, at least 50% of the high content of inducing metal that accumulates in the nuclei of each tumour, is bound by the nucleoli. The remainder is distributed approximately equally between the nuclear sap and chromatin.**

It was shown previously (Heath and Webb, 1967) that in primary rhabdomyosarcomata that have been induced in rats by intramuscular implantation of powdered metallic cobalt, cadmium or nickel, ions of the inducing metals are incorporated and bound intracellularly. Since the implanted metals dissolve and are eliminated slowly from the injection site, the contents of inducing metals in these tumours are variable. Irrespective of these variations, however, a common pattern is observed in the intracellular location of each of the cations, the major portion (70–90%) of which is associated with the nucleus. In further work, the results of which are summarized in this paper, the distribution within the nucleus of these bound cations has been investigated.

### MATERIALS AND METHODS

*Chemicals.*—Tris (Trizma Base, reagent grade) and DNase (Type I; electrophoretically purified, RNase-free) were obtained from Sigma Chemical Co., London, and sucrose (RNase-free) from Schwarz/Mann Division of Becton-Dickinson Co., Orangeburg, N.Y. All other chemicals were of Analar grade. Calcium chloride was prepared as a 15 mmol/l stock solution, and standardized by atomic absorption.

*Analytical methods.* — Phosphorus was measured by the method of Berenblum and Chain (1938) and RNA and DNA by the methods of Mejbaum (1939) and Burton (1956) respectively, with yeast RNA (C grade) and salmon sperm DNA (A grade; California Corp. for Biochemical Research, Los Angeles, U.S.A.) as standards.

The cations $Ni^{2+}$, $Co^{2+}$ and $Cd^{2+}$ were determined in wet-ashed samples (Heath and Webb, 1967) by atomic absorption with a Perkin–Elmer Model 303 spectrophotometer, the instrument being used in conjunction with a recorder-readout accessory at a scale expansion of $\times 3$ or $\times 10$.

*Induction of primary rhabdomyosarcomata by metallic cobalt, cadmium and nickel.*—These tumours were induced as described previously (Heath and Webb, 1967).

*Isolation of nuclei, nucleoli and chromatin.*—The experimental procedures were based on the methods of Sabatani *et al.* (1962), Muramatsu, Smetana and Busch (1963), DiGirolamo, Henshaw and Hiatt (1964), Dahmus and McConnell (1969) and Culp and Brown (1970). The rats were killed by cervical dislocation and the excised tumour tissue, freed from any necrotic material, was collected in medium A (0·25 mol/l sucrose, 3 mmol/l $CaCl_2$, 50 mmol/l Tris-HCl buffer, pH 7·6; DiGirolamo *et al.*, 1964) at 0°. The combined tissue was weighed, and homogenized in 5 vols medium A in a mechanical homogenizer with a loosely-fitting Teflon pestle, rotated at 1425

rev/min. The supernatant fraction was decanted from the residual tissue (20–25% of the initial weight), which was resistant to further homogenization, and strained first through 4 layers of surgical gauze then through nylon gauze. A measured volume of the filtrate was centrifuged for 10 min at 600*g* and the sedimented material re-suspended in 10 vols medium B (2·3 mol/l sucrose, 1·5 mmol/l $CaCl_2$, 10 mmol/l Tris-HCl buffer, pH 7·6) (DiGirolamo *et al.*, 1964). This suspension was homogenized briefly (about 30 seconds) in an ultra-Turrax homogenizer to remove cytoplasmic fragments that otherwise remained attached to the nuclei, and centrifuged for 45 min at 44,000*g*. The thick gelatinous layer at the top of each tube was removed with a spatula, and the remainder of the supernatant fraction syphoned off from the pellet. The inner walls of the centrifuge tubes were wiped with cotton swabs, the pellets re-suspended in medium A (1·5 vols) and the suspension centrifuged for 10 min at 12,000*g*. This procedure gave excellent preparations of nuclei, apparently free from cytoplasmic contamination, although the yields were low, since many nuclei remained enmeshed in the gelatinous layer that was discarded after centrifugation in medium B as described above. Thus on the basis of the values previously reported (Heath and Webb, 1967) for the intracellular distribution of $Ni^{2+}$ in primary nickel - induced rhabdomyosarcomata, the yields of nuclei from these tumours, as calculated from the fraction of the total $Ni^{2+}$ of the tissue homogenate that was recovered in the final preparation, was about 15%.

In experiments in which only the nucleoli were separated from the isolated nuclei, the latter were re-suspended in a suitable volume (usually 10 ml) of medium D (0·25 mol/l sucrose, 25 mmol/l KCl, 3·3 mmol/l $CaCl_2$ and 50 mmol/l Tris-HCl buffer, pH 7·8) (Culp and Brown, 1970) and the suspension treated at 0° with three 15-second pulses of ultrasonic vibrations in M.S.E. machine, operated at 1·5 A.* This total period of 45 seconds usually was optimal for the maximum disintegration of the nuclei and recovery of nucleoli. The resulting suspension was layered over medium D, in which the sucrose concentration had been increased to 0·88 mol/l, and centrifuged for 20 min at 4000 rev/min in the 3 × 20 rotor of a M.S.E. 65 centrifuge. The pellet was dispersed in fresh 0·88 mol/l sucrose medium, the suspension centrifuged at 800*g* for 15 min in the Sorvall SS 17 centrifuge, and the nucleoli again suspended in this medium. For further purification of the nucleoli this suspension was centrifuged briefly (1–2 min) at 200*g* and the supernatant fraction then centrifuged for a further 10 min at 800*g*. The purification of the preparation was followed by microscopy, and checked by the examination of methanol-fixed smears stained either with methyl green and pyronin, azure C (Murumatsu *et al.*, 1963) or by the $Zn^{2+}$-dithizone method (Studzinski, 1965).

For the removal of DNA from the nucleoli the latter were kept at 0° in a solution of DNase (100 $\mu$g/ml) in 0·25 mol/l sucrose, 50 mmol/l Tris-HCl buffer, pH 7·4 and 7·5 mmol/l $MgCl_2$) (Murumatsu *et al.*, 1963) for various times, and then layered over 0·88 mol/l sucrose. The nucleoli were recovered by centrifugation for 20 min at 800*g*.

The above procedure was used also for the isolation and purification of nucleoli from nuclei that, for ultrasonic disintegration, were suspended in rat liver cell sap, supplemented with 2 mmol/l calcium acetate, to prevent possible degradation by endogenous RNase (Matsuhisa *et al.*, 1970).

When chromatin as well as nucleoli was isolated, the tumour cell nuclei were re-suspended in a suitable volume (7·5–10·0 ml) of RSB saline (Zimmerman *et al.*, 1969). A portion of the suspension was treated with ultrasonic vibrations as described above, and then centrifuged for 5 min at 2500*g*. Chromatin was recovered from the supernatant fraction by centrifugation for 10 min at 10,000*g*, and purified as described by Dahmus and McConnell (1969). The nucleolar pellet was resuspended in RSB to a volume of 5·5 ml and 5 ml of this suspension mixed with an equal volume of medium C (1·76 mol/l sucrose, 6·6 mmol/l $CaCl_2$ and 20 mmol/l Tris-HCl buffer, pH 7·6). This was centrifuged for 15 min at 200*g*. The

* To investigate the possible contamination of the subfractions of the nuclei by metallic ions derived from the probe of the ultrasonic disintegrator, the latter was operated for 2 hours in 0·9% (w/v) NaCl and RSB (5 ml). After acidification of these solutions at 60–70° with 12N HCl to a final concentration of 1N, neither $Co^{2+}$, $Ni^{2+}$ nor $Cd^{2+}$ were detected by atomic absorption.

crude nucleolar fraction was either analysed at this stage, or was purified further as outlined above.

In one experiment, fractionation of cadmium-induced tumours preserved by deep freezing, was attempted. The nuclei that were obtained from this tissue in extremely poor yield were morphologically abnormal, but gave an apparently satisfactory preparation of nucleoli.

### RESULTS

On fractionation of the isolated nuclei from primary tumours induced by metallic nickel and cobalt, the recovery of nuclear $Ni^{2+}$ and $Co^{2+}$ in all fractions (*i.e.* crude chromatin, nuclear sap and nucleoli) averaged 93% and 97%, respectively. Recovery of $Cd^{2+}$ in the subfractions of the nuclei from the primary cadmium-induced tumour was less satisfactory (81%). Although, in seven different preparations of nucleoli from primary nickel-induced tumours the content of $Ni^{2+}$ varied with that in the initial suspension of nuclei, the percentage recovery of nuclear $Ni^{2+}$ in the nucleoli was reasonably constant (mean 53%; range 41·4–62·8%). This recovery seemed unaffected by the composition of the medium that was used to suspend the nuclei for ultrasonic disintegration. Corresponding values for the contents of nuclear $Co^{2+}$ and $Cd^{2+}$ in the nucleoli of primary tumours, induced by metallic cobalt and cadmium, were 52% and 72%, respectively. These figures must be considered to be very approximate since, although the conditions of ultrasonic treatment were standardized to give optimal breakage of nuclei with minimal loss of nucleoli, the yields of the latter were by no means quantitative. Also the crude preparations, as initially isolated, were contaminated with some whole nuclei and other, unidentified, material. In those fractionations of nuclei from the three primary tumours that were done in the RSB medium, to enable the chromatin to be recovered, the remainder of the cation was found to be distributed approximately equally between the sedimented material (chromatin) and the soluble components (nuclear sap).

Purified preparations of isolated nucleoli from each of the primary tumours varied in size but, characteristically, contained on average about 3 times as much DNA as RNA (DNA/RNA 2·62–4·56). This ratio is higher than that given by Muramatsu *et al.* (1963) for nucleoli of the Walker carcinosarcoma, but is similar to that reported for liver nucleoli (Busch, Byvoet and Smetana, 1963). Most of the nucleolar DNA was removed by digestion of the particles with DNase, and even a brief (1 hour) treatment of a nucleolar preparation from the nickel-induced tumour with the enzyme at 0° reduced the DNA content by 56·5%. This removal of DNA was accompanied by some loss (19·3%) of $Ni^{2+}$, but only a small decrease (2·9%) in the content of RNA. Thus, although some $Ni^{2+}$ appeared to be bound to the "nucleolar" DNA, in terms of nucleic acid content the concentration of the cation in this preparation of nucleoli was increased from 25·1 to 34·6 $\mu$g $Ni^{2+}$/mg nucleic acid P by treatment with DNase. Previous observations (Heath and Webb. 1967) have shown that binding of $Ni^{2+}$ by nuclear RNA of nickel-induced primary tumours is much greater than by DNA. Expression of metal concentrations of nuclear sub-fractions in terms of nucleic acid contents, however, may be misleading, in so far as it may over-emphasize the importance of these components in cation binding. This is illustrated by the results, obtained with a preparation of nuclei from primary nickel-induced tumours (Table I), which show the distribution of $Ni^{2+}$ amongst the nuclear subfractions expressed both as a percentage of the total nuclear $Ni^{2+}$, and in terms of nucleic acid content. Nevertheless, in the absence of a more suitable and convenient parameter, the total nucleic acid P was useful as a reference standard to demonstrate that the content of $Ni^{2+}$ (Table I), $Co^{2+}$ or $Cd^{2+}$ in the

TABLE I.—*Distribution of $Ni^{2+}$ in the Subfractions of Nuclei from Primary, Nickel-induced Rhabdomyosarcomata*

| Fraction | Per cent of total $Ni^{2+}$ in nuclei* | $\mu g$ $Ni^{2+}$/mg nucleic acid P |
|---|---|---|
| Nuclei | — | 29·2 |
| Nuclear sap† | 14·3 | 39·6 |
| Chromatin† | 16·3 | 24·2 |
| Crude nucleoli | 62·8 | 41·7 |
| Purified nucleoli | — | 58·6 |

* Percentage of nuclear $Ni^{2+}$ recovered in all subfractions = 93·4.

† In these fractions $\Sigma$RNAP + DNAP was significantly less than the content of total P.

nucleolar fraction of the corresponding metal-induced tumour increased with further purification.

### DISCUSSION

The present fractionation studies show that ions of the inducing metals, which are associated with the nuclei of primary metal-induced rhabdomyosarcomata (Heath and Webb, 1967), are bound by components of the nuclear sap, by the chromatin and, particularly, by the nucleoli. It is thus possible that the nuclear uptake of these cations during growth and their incorporation into the nucleic acids during biosynthesis could affect the conformation and biological activity of both DNA and RNA. From the results of the following paper (Weinzierl and Webb, 1972) it is apparent that the specificity of the carcinogenic metals is not determined by their solubilities in biological fluids, since other, non-carcinogenic, metals also dissolve under these conditions. It seems, therefore, that the specificity may lie in the subsequent effects of the dissolved metals, particularly when the ions are incorporated intracellularly. Thus the molecular geometry of the cation complexes may be an important factor. In this connection, Beach and Sunderman (1970) have shown that a chromatin–RNA polymerase complex, when isolated from the nuclei of liver tissue of rats treated with nickel carbonyl, contains $Ni^{2+}$ and has a much lower enzymic activity than that of a similar preparation from control animals. This inhibition seems to be related to the incorporation of $Ni^{2+}$ into the enzyme complex, since the addition of similar, or slightly greater amounts of $Ni^{2+}$ (as $Ni(CO)_4$ or $NiCl_2$) directly to the control system *in vitro* was found to be without effect upon enzyme activity. Also, it is significant that the method used by Beach and Sunderman (1970) for the isolation of the polymerase complex would yield a preparation of nucleoli as well as chromatin.

As discussed previously (Heath and Webb, 1967), it is difficult to assess the significance of the distribution of ions of the inducing metals amongst the subcellular components of well-developed tumours. The possibility that a similar pattern of distribution may be common to (regenerating) cells in the neighbourhood of the metal implants soon after implantation, and to established tumours, derives some support from recent observations (Webb and Weinzierl, 1972) on the intracellular location of $^{63}Ni^{2+}$ in cells of the C575/1P strain of mouse dermal fibroblasts after growth for short periods *in vitro* in the presence of complexes of $^{63}Ni^{2+}$ with both proteins and small, diffusible molecules. This work has shown that $^{63}Ni^{2+}$ from these complexes is incorporated intracellularly and that, irrespective of the nature of the carrier, in general the largest amount is bound by the nuclei. Moreover, about half of the $Ni^{2+}$ incorporated into the nuclei of the cultured cells is associated with the nucleoli. This is very similar to the average value of 53% found in the present work for the nucleolar content of the nuclear cation in primary nickel-induced tumours. The affinity of the carcinogenic metals for the nucleoli, both in the primary tumours and, probably in cultured cells, coupled with previous observations on the presence of "persistent" nucleoli in the cytoplasm of chick fibroblasts, after treatment in culture with $Co^{2+}$ (Heath, 1954) suggests the possibility that these ions may affect the processing of the ribosomal precursor RNA (see *e.g.* Darnell, 1968).

Although Heath and Webb (1967) have shown that nucleic acids from the nuclei of primary nickel-induced tumours contain $Ni^{2+}$, and that binding of the cation by nuclear RNA is greater than by DNA, it cannot be assumed, however, that the nucleolar accumulation of $Ni^{2+}$ occurs predominantly in RNA. The selective staining of nucleoli by the $Zn^{2+}$-dithizone procedure of Studzinski (1965) for example, is due to the presence in these organelles of a protein of high binding-affinity for $Zn^{2+}$. Such a protein would be expected also to bind $Cd^{2+}$ and other cations (*e.g.* $Co^{2+}$ and $Ag^{+}$; Tandler, 1953, 1954). Even if the interaction of metallic ions occurs mainly with proteins of the nucleus and/or nucleolus, however, the resulting modifications in structure could affect nucleic acid function by de-repression, as discussed by MacGillivray and Paul (1971).

This work was done with the support of the Medical Research Council (M. Webb) and Cancer Research Campaign (J. C. Heath). T. Hopkins is grateful to the Medical Research Council for the award of a Sandwich Course Studentship.

The technical assistance of Miss Diane Jackson and Mr G. Payton in the biochemical work, and Miss Angela Orledge with the induction of the primary tumours, is gratefully acknowledged.

### REFERENCES

Beach, D. J. & Sunderman, F. W. (1970) Nickel Carbonyl Inhibition of RNA Synthesis by a Chromatin-RNA Polymerase Complex from Hepatic Nuclei. *Cancer Res.*, **30**, 48.

Berenblum, I. & Chain, E. (1938) Studies on the Colorimetric Determination of Phosphate. *Biochem. J.*, **32**, 286.

Burton, K. (1956) A Study of the Conditions and Mechanism of the Diphenylamine Reaction for the Colorimetric Determination of Deoxyribonucleic Acid. *Biochem. J.*, **62**, 315.

Busch, H., Byvoet, P. & Smetana, K. (1963) The Nucleolus of the Cancer Cell: a Review. *Cancer Res.*, **23**, 313.

Culp, L. A. & Brown, G. M. (1970) RNA-methylases from Rat Liver Nucleoli. *Archs Biochem. Biophys.*, **137**, 222.

Dahmus, M. E. & McConnell, D. J. (1969) Chromosomal Ribonucleic Acid of Rat Ascites Cells. *Biochemistry*, **8**, 1524.

Darnell, J. E. (1968) Ribonucleic Acids from Animal Cells. *Bact. Rev.*, **32**, 262.

Digirolamo, A., Henshaw, E. C. & Hiatt, H. H. (1964) Messenger Ribonucleic Acid in Rat Liver Nuclei and Cytoplasm. *J. molec. Biol.*, **8**, 479.

Heath, J. C. (1954) The Effect of Cobalt on Mitosis in Tissue Culture. *Expl. Cell Res.*, **6**, 311.

Heath, J. C. & Webb, M. (1967) Content and Intracellular Distribution of the Inducing Metal in the Primary Rhabdomyosarcomata Induced in the Rat by Cobalt, Nickel and Cadmium. *Br. J. Cancer*, **21**, 768.

MacGillivray, A. H. & Paul, J. (1971) Control of Transcription of Chromatin Deoxyribonucleic Acid. *Biochem. J.*, **124**, 40P.

Matsuhisa, T., Higashi, K., Gotoh, S. & Sakamoto, Y. (1970) Changes in the Nucleotide Compositions of Nucleolar 45S RNA of Azo-dye Induced Hepatoma. *Cancer Res.*, **30**, 162.

Mejbaum, W. (1939) Estimation of Small Amounts of Pentose, Especially in Derivatives of Adenylic Acid. *Hoppe-Seyler's Z. physiol. Chem.*, **258**, 117.

Muramatsu, M., Smetana, K. & Busch, H. (1963) Quantitative Aspects of Isolation of Nucleoli of the Walker Carcinosarcoma and Liver of the Rat. *Cancer Res.*, **23**, 510.

Sabatani, A., de Kloet, S. R., Allfrey, V. G. & Mirsky, A. E. (1962) Isolation of a Nuclear RNA Fraction Resembling DNA in its Base Composition. *Proc. natn. Acad. Sci. U.S.A.*, **48**, 471.

Studzinski, G. P. (1965) Selective Binding of Zinc by Basic Proteins of the HeLa Cell Nucleus. *J. Histochem. Cytochem.* **13**, 365.

Tandler, C. J. (1953) The Use of Cobalt Acetate in the Histochemical Technic for Acid Phosphatase. *J. Histochem. Cytochem.*, **1**, 151.

Tandler, C. J. (1954) An Argentaffin Component of the Nucleolus. *J. Histochem. Cytochem.*, **2**, 165.

Webb, M. & Weinzierl, S. M. (1972) Uptake of $^{63}Ni^{2+}$ from its Complexes with Proteins and other Ligands by Mouse Dermal Fibroblasts *in vitro*. In preparation.

Weinzierl, S. M. & Webb, M. (1972) Interaction of Carcinogenic Metals with Tissue and Body Fluids. In preparation.

Zimmerman, E. F., Hackney, J., Nelson, P. & Arias, I. M. (1969) Protein Synthesis in Isolated Nuclei and Nucleoli of HeLa Cells. *Biochemistry*, **8**, 2636.